# Precise higher-order reflectivity and morphology models for early diagnosis of diabetic retinopathy using OCT images

**DOI:** 10.1038/s41598-021-83735-7

**Published:** 2021-02-25

**Authors:** A. Sharafeldeen, M. Elsharkawy, F. Khalifa, A. Soliman, M. Ghazal, M. AlHalabi, M. Yaghi, M. Alrahmawy, S. Elmougy, H. S. Sandhu, A. El-Baz

**Affiliations:** 1 BioImaging Laboratory, Department of Bioengineering, University of Louisville, Louisville, KY USA; 2 Electrical and Computer Engineering Department, Abu Dhabi University, Abu Dhabi, UAE; 3 Faculty of Computers and Information, Mansoura University, Mansoura, Egypt; 4 Department of Ophthalmology and Visual Sciences, University of Louisville, Louisville, USA

**Keywords:** Engineering, Biomedical engineering

## Abstract

This study proposes a novel computer assisted diagnostic (CAD) system for early diagnosis of diabetic retinopathy (DR) using optical coherence tomography (OCT) B-scans. The CAD system is based on fusing novel OCT markers that describe both the morphology/anatomy and the reflectivity of retinal layers to improve DR diagnosis. This system separates retinal layers automatically using a segmentation approach based on an adaptive appearance and their prior shape information. High-order morphological and novel reflectivity markers are extracted from individual segmented layers. Namely, the morphological markers are layer *thickness* and *tortuosity* while the reflectivity markers are the 1st-order reflectivity of the layer in addition to local and global high-order reflectivity based on *Markov-Gibbs random field (MGRF)* and *gray-level co-occurrence matrix (GLCM)*, respectively. The extracted image-derived markers are represented using cumulative distribution function (CDF) descriptors. The constructed CDFs are then described using their statistical measures, i.e., the 10th through 90th percentiles with a 10% increment. For individual layer classification, each extracted descriptor of a given layer is fed to a support vector machine (SVM) classifier with a linear kernel. The results of the four classifiers are then fused using a backpropagation neural network (BNN) to diagnose each retinal layer. For global subject diagnosis, classification outputs (probabilities) of the twelve layers are fused using another BNN to make the final diagnosis of the B-scan. This system is validated and tested on 130 patients, with two scans for both eyes (i.e. 260 OCT images), with a balanced number of normal and DR subjects using different validation metrics: *2-folds*, *4-folds*, *10-folds*, and *leave-one-subject-out (LOSO)* cross-validation approaches. The performance of the proposed system was evaluated using *sensitivity*, *specificity*, *F1-score*, and *accuracy* metrics. The system’s performance after the fusion of these different markers showed better performance compared with individual markers and other machine learning fusion methods. Namely, it achieved $96.15\%$, $99.23\%$, $97.66\%$, and $97.69\%$, respectively, using the LOSO cross-validation technique. The reported results, based on the integration of morphology and reflectivity markers and by using state-of-the-art machine learning classifications, demonstrate the ability of the proposed system to diagnose the DR early.

## Introduction

Multiple eye diseases that are major public health threats are asymptomatic or minimally symptomatic in their early states. These include diabetic retinopathy (DR), age-related macular degeneration (AMD), glaucoma, and choroidal neovascularization (CNV), all of which are potentialy blinding. Because of the minimal symptomatology in their early stages, these diseases are typically detected on routine examinations. This paper concentrates on DR, which is a chronic disease that causes progressive microvasculopathy and retinal ischemia. According to Saeedi et al.^1^, the number of DR patients will increase from 463 million patients (31 million in the United States) in 2019 to 578 million (34.4 million in the United States) by 2030 and 700 million (36 million in the United States) by 2045. Moreover, in a recent study from the Centers for Disease Control and Prevention (CDC)^2^, DR prevalence in the United States was estimated at 4.1 million, of whom 899, 000 had visual impairments. Detecting DR in its early stages is critical. Patients who present only once they become symptomatic frequently often have advanced disease with multiple, potentially blinding structural complications, such as proliferative diabetic retinopathy, neovascular glaucoma, and/or tractional retinal detachment. Therefore, it is important to detect DR early in order to prevent irreversible vision loss.

Multiple different imaging modalities are used by ophthalmologists to assess the retina, such as optical coherence tomography (OCT), OCT angiography (OCTA), fundus photography (FP), and fluorescein angiography (FA). Each modality has the virtue of revealing different features of the retina.

Significant research into retinal imaging modalities has already been conducted in the last few years to detect the pathologies of the eye early before causing any damage to the blood vessels of the retina and leading to vision loss. Some use FP, which takes a lot of time to distinguish morphological modifications in the optic disc and macula through examination. Priya et al.^3^ proposed a system to diagnose the grades of DR in FP. Their system consisted of four steps: *1)* A pre-processing step was applied to enhance the FP image. The latter was an adaptive histogram equalization to increase the contrast, a discrete wavelet transform (DWT) to reduce the image dimension and the computing time, and matched filter response (MFR) for noise reduction. *2)* Blood vessels and exudates were segmented using fuzzy C-means clustering. *3)* Six features were extracted from the segmented image, namely area, half area, arc length, radius, diameter, and center angle. *4)* Three different classification methods (support vector machine (SVM), Bayes classifiers, and probabilistic neural network (PNN)) were applied to these features to detect and grade DR in the FP. The accuracy of the reported three classifiers was $89.6\%$, $94.4\%$, and $97.6\%$ for Bayes classifiers, PNN, and SVM, respectively. A similar study^4^ presented an automatic diagnostic system of DR by extracting the blood vessels and hemorrhages from FP, then a *gray-level co-occurrence matrix (GLCM)* was constructed and statistical features were extracted from this matrix. Finally, SVM was used to make the final diagnosis in which the reported accuracy was $82.35\%$. In another study, an automatic system based on mathematical morphology has been presented by detecting macular hard exudates in FP^5^. The hard exudates are extracted by taking the complement of the extended minima transform. Then, the scan was classified as diabetic maculopathy (DM) if it has one or more hard exudates. Finally, the system grades the DM as clinical significant diabetic macular edema (CSME) or clinically non-significant diabetic macular edema (Non-CSME) based on the extent to which the hard exudates involve the fovea. The precision, recall, and area under the curve (AUC) of the system were $86.67\%$, $100\%$, and $97.06\%$, respectively. A similar work^6^ proposed a computer assisted diagnosis (CAD) system to detect, and classify the different grades of DM and DR. The sensitivity and specificity of diagnosing the DM were $96.46\%$ and $98.56\%$, respectively while the overall accuracy of grading DR was $94.33\%$. Another automatic system based on FP was developed by Rahim et al.^7^. The system extracted three different features from the extracted exudates in the whole scan and macula, i.e., six features in total. The latter were mean, standard deviation, and area of the extracted regions. The six features were fed to four different classifiers individually. The reported results were accuracy of $70\%$ ($93\%$, $93\%$, $75\%$), sensitivity of $45.28\%$ ($92.45\%$, $86.79\%$, $60.38\%$), and specificity of $97.87\%$ ($93.62\%$, $100\%$, $91.49\%$) for SVM using a polynomial kernel (SVM using a radial basis function kernel, K-nearest neighbor (KNN), Naïve Bayes). Other studies have applied deep learning on FP to diagnose DR, e.g. Hemanth et al.^8^. The authors developed a deep convolution neural network (CNN) to detect and classify DR in FP. Before input to the CNN, a histogram equalization and a contrast limited adaptive histogram equalization were applied to each channel of the image to enhance the image contrast, then the three modified channels were concatenated again. The reported sensitivity, precision, F1-score, specificity, geometric mean of sensitivity and specificity, and accuracy were $94\%$, $94\%$, $94\%$, $98\%$, $95\%$, and $97\%$, respectively. In another study, a deep-based neural network was presented to detect the five grades of the DR^9^. dense color scale-invariant feature transform (DColor-SIFT) was applied to select the point of interest (PoI), followed by a gradient location-orientation histogram (GLOH) to enhance the performance of the classification. The dimension of this descriptor was decreased using principal component analysis (PCA). Finally, three deep layers were fed with this descriptor to learn new features and then to diagnose the stages of the DR. The sensitivity, specificity, and AUC of the system were $92.18\%$, $94.5\%$, and $92.4\%$, respectively. Other studies that use FP for automated diagnosis are also provided for reference^10–14^.

Other studies have used OCT and OCTA modalities to diagnose retinal diseases, as they are non-invasive techniques that produce a cross-sectional or volumetric view of the retina and blood vessels, respectively. Alam et al.^15^ proposed an automatic CAD system to classify normal versus DR and also normal versus the grades of DR in the OCTA scans using an SVM. The developed system extracted six features from the OCTA scans, namely blood vessel caliber, blood vessel tortuosity, blood vessel density, foveal avascular zone (FAZ) area, vessel perimeter index, and FAZ contour irregularity. Their experiments presented the results of each feature individually as well as a fusion of all features combined. While, the most predictive feature was blood vessel density, features fusion gave the best performance of all experiments. The reported accuracies of the fusion features for normal versus DR and normal versus the grades of DR were $94.41\%$ and $92.96\%$, respectively. In another study^16^, the authors developed an extension CAD system of their previous work^17^ to detect DR in the 3D volume of the OCT by extracting three features, namely histogram of oriented gradients (HOG), PCA, and local binary pattern (LBP). The system used different classification methods fed with these features individually, some, or all of them in which the best sensitivity and specificity reached were $87.5\%$ and $87.5\%$, respectively using an SVM with linear kernel fed with the histogram of LBP using PCA. This study has many limitations, including sub-optimal performance and a lack of layer segmentation. A modified VGG16-based CAD system was presented by Ibrahim et al.^18^ to diagnose DM, CNV, and drusenoid disorders in the OCT volumes by fusing hand-crafted features from region of interest (RoI) with the learned features extracted from CNN in which the reported sensitivity, specificity, and accuracy of this study were $99.4\%$, $98.2\%$, and $98.8\%$, respectively. Another study by Ghazal et al.^19^ presented a CNN CAD system to diagnose DR in the OCT B-scans. Their system consisted of three stages: *1)* Each B-scan is divided into five regions, namely distal temporal, temporal, central, nasal, and, distal nasal. *2)* Seven different CNNs were trained, one per region plus two using transfer learning on nasal and temporal regions only. *3)* The seven CNN outputs were passed, singly or in combination, to SVM with the linear kernel to make the final diagnosis. The reported accuracy of the developed system was $94\%$ when using transfer learning and two regions (nasal and temporal). Other studies presented a CAD system to detect the grades of DR that used a combination of the two modalities^20^. The outputs of the two modalities are fused with clinical, demographic, data and fed into a random forest (RF) classifier in which the reported accuracies for detecting the DR and the grades of the DR were $98.2\%$ and $98.7\%$, respectively. Other studies have also utilized OCT with varying results^21–23,23–36^.

In spite the plethora of diagnostic systems using OCT B-scans, there are some limitations apparent in the current literature, such as *1)* using inaccurate (i.e., threshold-based) or manual segmentation, *2)* unnecessary markers unrelated to DR diagnosis, and *3)* low diagnostic performance. Therefore, in this paper, a novel CAD system to detect DR using OCT B-scans is proposed to partially overcome these limitations. The first step of the developed system is to segment the twelve layers of a given scan based on an atlas-based approach. Then, four different markers are extracted from each segmented layer and fed separately into a machine learning (ML) classifier. Finally, the results of the classifiers are fused using two backpropagation neural networks (BNN) to make the final diagnosis. This paper provides additional development of our previous work^37,38^, with the following contributions: *1)* We investigate and diagnose each layer of the OCT’s twelve layers individually as the local diagnosis of each layer is obtained by fusing the diagnosis of each marker individually using BNN. The global decision is made by fusing the diagnosis of each individual layers using another BNN. *2)* We propose local and global higher-order reflectivity markers based on a Markov-Gibbs random field (MGRF) model and GLCM. *4)* A more descriptive approach (involving percentiles of the cumulative distribution function (CDF)) is applied to the extracted markers. *5)* The performance of the diagnostic system using OCT modality is enhanced.

**Figure 1:**
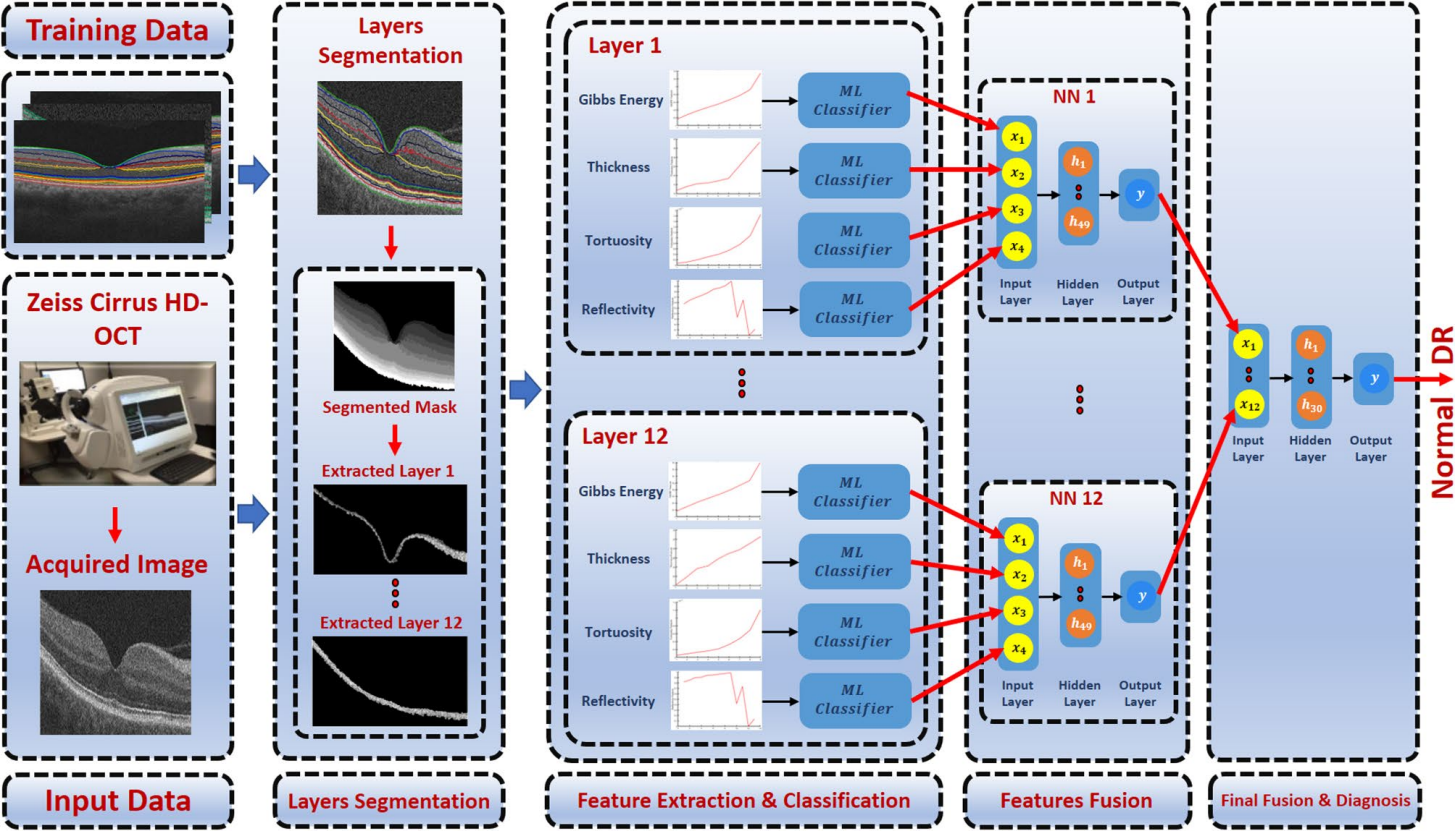
Schematic illustration of the pipeline of the proposed system for DR diagnosis using OCT images.

## Methods

The proposed CAD system to diagnose the DR disease based on the central B-scan (through the foveal pit) of the volumetric OCT scans is depicted in Fig. 1. This proposed system consists of three steps: (1) Detection and segmentation of the twelve retinal layers within the B-scan use our previously developed appearance-based approach^37^. (2) Descriptive markers are extracted including a higher-order reflectivity metrics that combine both local and global terms estimated using high-order MGRF and GLCM matrix, in addition to morphological features (i.e., thickness, tortuosity) from each segmented layer, which are fed separately to an SVM classifier. (3) The four classifiers’ results are fused using a backpropagation neural network (BNN) to determine the final diagnosis of that layer. Lastly, another BNN is used to fuse the diagnostic results of the twelve layers to determine the final diagnosis of the B-scan. More details of the segmentation technique, marker extraction, and the employed machine learning classifier are presented in “Retinal layers segmentation”, “Image markers extraction”, and “Classification”, respectively.

**Figure 2:**
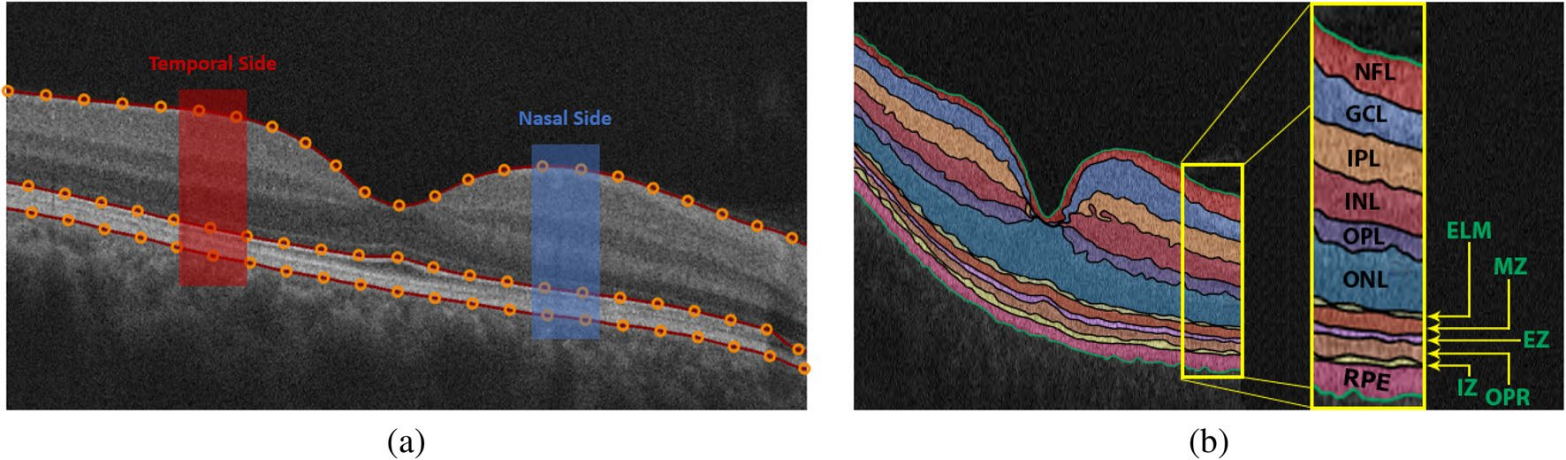
Illustrative example of the proposed segmentation approach: (**a**) detection of vitreous and choroid, and (**b**) the final twelve segmented layers.

### Retinal layers segmentation

**Figure 3:**
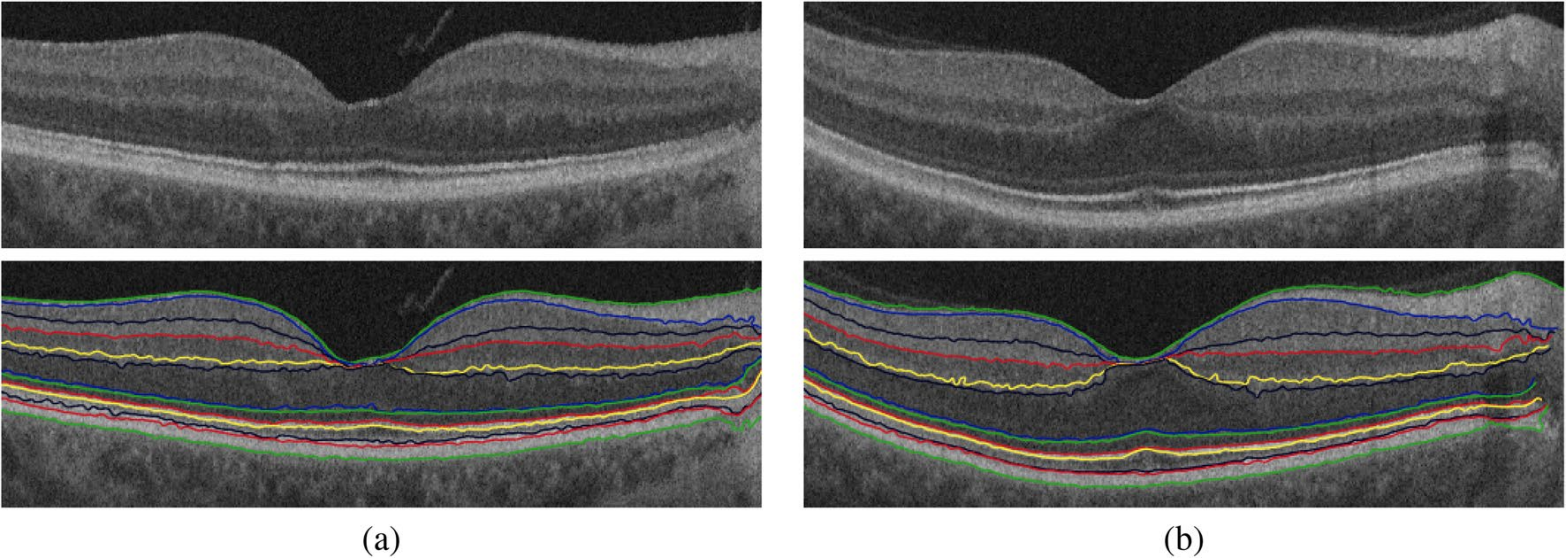
Sample example of the 12-layers segmentation for (**a**) a normal and (**b**) a DR case using our approach.

The retina proper and the retinal pigment epithelium, i.e. the tissue between the internal limiting membrane and Bruch’s membrane, as it appears in OCT imaging, can be parcellated into twelve layers. In order from innermost to outermost, these are the nerve fiber layer (NFL), ganglion cell layer (GCL), inner plexiform layer (IPL), inner nuclear layer (INL), outer plexiform layer (OPL), outer nuclear layer (ONL), external limiting membrane (ELM), myoid zone (MZ), ellipsoid zone (EZ), outer photoreceptor segments (OPR), interdigitation zone (IZ), and retinal pigment epithelium (RPE) (Fig. 2). Therefore, this step aims to accurately segment the twelve retinal layers of the OCT image. To reach this goal, a segmentation approach has been developed that extracts the layers automatically from the fovea using shape prior information^37^. Before segmenting the layers, the boundary of the vitreous and choroid are detected using a multiresolution edge detector as shown in Fig. 2a. Then, a non-linear registration with thin-plate splines^39^ was used to align the retina outline with the shape database, constructed from a set of manually segmented B-scans. The latter were outlined by a retina specialist. After that, each pixel of a given OCT B-scan to be segmented is initially classified as belonging to one of the twelve layers. This is done using the relative pixel intensity combined with an adaptive model that utilizes the OCT images and their manually segmented maps in the shape database and is adaptively updated for each input scan. Finally, the final segmentation is obtained using spatial smoothing and topological constraints (e.g., the NFL layer must lie above the GCL layer). Mathematically, this is described using a joint probability model given by Eq. (1). An example of the segmentation results using our approach for a normal and DR retinas is shown in Fig. 3.

1 $$\begin{aligned} P(g,l)=P(g|l)P_{s}(l) P_{V}(l) \end{aligned}$$

where

- *g* and *l* are the intensity value of the input-aligned image and its label map, respectively.
- *P*(*g*|*l*) is the intensity probability model, estimated using the modified expectation maximization (EM) algorithm^40,41^.
- $P_{s}(l)$ is the shape probability that is adaptively constructed during segmentation using the manually segmented grayscale images and their respective maps as well as the input-aligned OCT image^37^.
- $P_{V}(l)$ is the spatial smoothing probability term described by a second-order Markov-Gibbs random filed (MGRF) model^41^ using the 8-pixel-connectivity and analytical potentials^42,43^.

**Figure 4:**
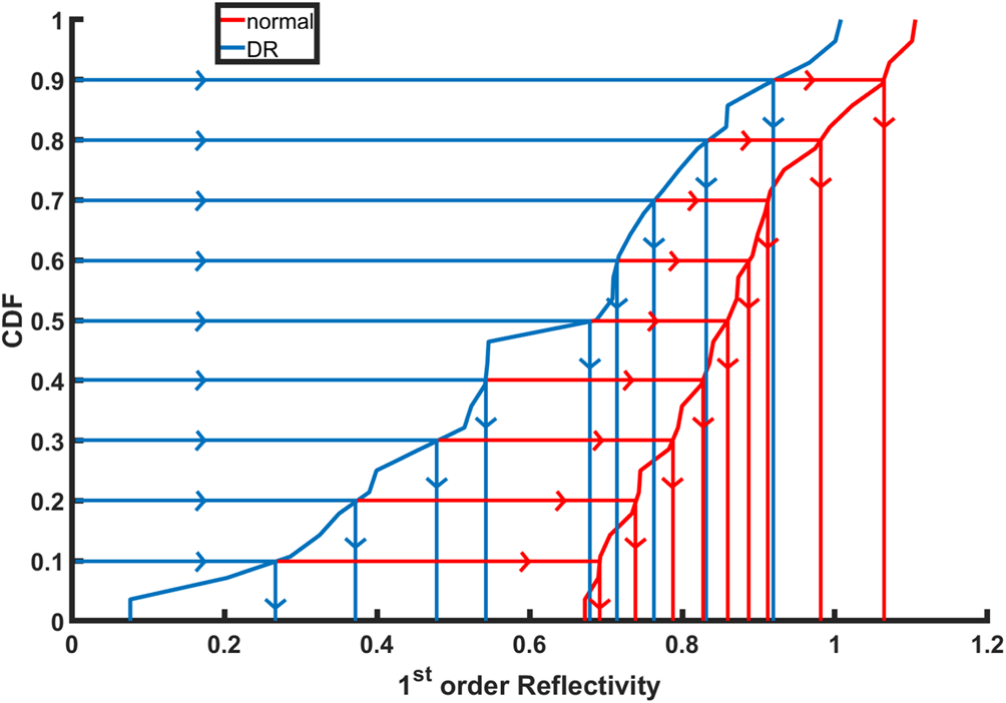
An illustrative example of the estimated CDF percentile feature of the 1st-order reflectivity for a normal and a DR case at the NPL layer.

### Image markers extraction

The second critical and important step of the proposed system is the precision modeling of the image features that describe each of the segmented layers of a given OCT B-scan. The more accurate the descriptor is, the more accurate the diagnosis is. Therefore, the segmented layer is represented carefully by a set of discriminant features using higher-order morphological and novel reflectivity features. Details are given below. A pre-processing step is conducted before feature extraction and representation to reduce contamination by outlier measurements, which are often found near the edges of the segmented B-scan. Namely, a predefined region of the OCT B-scan is selected while the position of the fovea is placed at the center. Then, the morphological and reflectivity features are measured at different bins of the segmented B-scan due to the different size of each layer, then the average value of these markers at each bin is computed. Finally, to better represent any of the derived markers, a compact representation is used. Namely, a cumulative distribution function (CDF) of the extracted marker values is first constructed, then only a set of nine statistical measures of the CDF descriptor is selected. Namely, the 10th through 90th percentiles, with a 10% increment, as shown in Fig. 4. This is applied to all markers except GLCM markers.

#### Reflectivity

The first image marker used in our system is reflectivity, which represents the intensity of the reflection of light off an individual layer. To precisely describe retinal layers and thus enhance the accuracy of our system, the reflectivity marker is not only represented with the traditional 1st-order term, but also with higher-order reflectivity terms that are less sensitive to scan noise. The 1st-order reflectivity is represented by the average pixel intensity in each of the predefined bins of each layer. Also, this marker is measured only on a predefined region at both the temporal and nasal sides because the innermost five layers vanish near the fovea, as shown in Fig. 2a.

**Figure 5:**
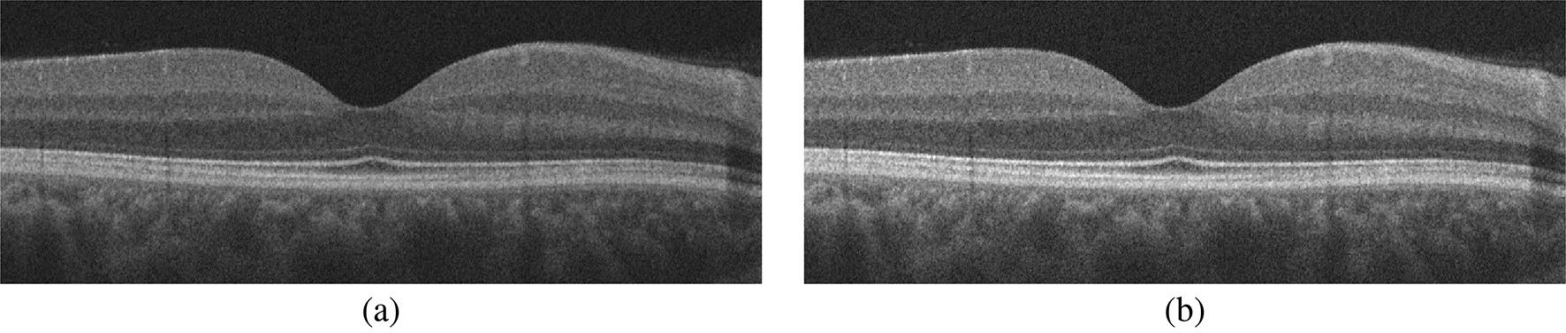
OCT scan normalization: (**a**) original image, and (**b**) normalized image.

Before estimating the reflectivity, a given OCT B-scan ($I_{in}$) is first normalized by $I_{n} =\frac{I_{in}-RV}{R_{12}-RV} \times 1000$, as the OCT pixel gray level is not an absolute metric of reflectivity since it depends on some external factors, such as pupil dilation, that affect image quality. For example, the retinal NFL in an eye that is insufficiently dilated may appear darker than in a fully dilated eye, even in the case where both eyes are of the same age and free of pathology. Therefore, a relative metric of reflectivity is used, where the reflectivity of the NFL and other layers is a fraction of the RPE reflectivity. It is standardized with respect to the RPE because that layer is typically preserved in early DR. Here, *RV* and $R_{12}$ are the average intensity of vitreous, and the average intensity of the RPE layer, respectively. An illustration of the normalization step is shown in Fig. 5.

Traditional reflectivity, however, may not precisely describe the OCT signal, especially in the diseased retina. Thus, we propose to add higher-order terms that have the ability to overcome scan noise and artifacts. Those terms represent both the local and global higher-orders reflectivity and are based on *Gibbs energy* derived from the co-occurrence matrix, and *GLCM*, respectively. To develop the local higher-order reflectivity model, each OCT image is considered as a sample of a Markov-Gibbs random field (MGRF) with a translation-invariant system of pairwise interactions. Before we describe the proposed model let us identify the following arithmetic symbols:

- Let $N = \{({\zeta }_{i},{\eta }_{i}), i=1,\ldots ,n\}$ be a finite set of (*x*, *y*)-offsets specifying neighborhood system of MGRF model.
- *R* is the 2D arithmetic grid of the 2D OCT image.
- The neighborhood system for pixel (*x*, *y*) for a giving offset $({\zeta }_{i},{\eta }_{i})$ is defined as follows: $\{((x+{\zeta }_{i},y+{\eta }_{i}), (x-{\zeta }_{i},y-{\eta }_{i})); ({\zeta }_{i},{\eta }_{i})\in N\}$.
- Let ${C}_{\zeta ,\eta }$ be a family of pairwise cliques $C_{\zeta ,\eta ;x,y} = ((x,y), (x+\zeta ,y+\eta ))$ with offset $(\zeta ,\eta )\in N$ in the interaction graph on *R*.
- Let *V* be a vector of Gibbs potentials for gray level co-occurrences in the cliques: ${V}^{T} = [{V}^{T}_{\zeta ,\eta } : (\zeta ,\eta ) \in N]$, where ${V}^{T}_{\zeta ,\eta }=[{V}_{\zeta ,\eta }(q,s) : (q,s) \in {Q}^{2}]$, *Q* is the number of grey levels in the OCT image.

A generic 2nd-order reflectivity-based MGRF on 2D lattice *R* is specified by the Gibbs probability distribution function:

2 $$\begin{aligned} P(g)=\frac{1}{Z} \exp \left( |R| {V}^{T}F(g) \right) \end{aligned}$$

where *Z* is the partition function, |*R*| is the cardinality of *R*, and *F*(*g*) is the vector of scaled empirical probability distributions of gray level co-occurrences over each clique family. To identify the MGRF model in Eq. 2, we have to estimate the clique potentials *V*. To achieve this goal, we use the analytical maximum likelihood estimator for the 2nd-order Gibbs potentials introduced in^42^:

3 $$\begin{aligned} V_{layer,\zeta ,\eta } (q,s)= \rho _{layer,\zeta ,\eta } [f_{layer,\zeta ,\eta } (q,s)-f_{layer} (q)f_{layer} (s)] \end{aligned}$$

where $f_{layer}(q)$ is an empirical marginal distribution of pixel intensities, $f_{layer,\zeta ,\eta }(q,s)$ is an empirical joint distribution of intensity co-occurrences. Thus, for each layer in the OCT images that energy of the 2nd-order reflectivity-based on the MGRF model can be calculated as follows:

4 $$\begin{aligned} E_{layer,\zeta ,\eta }=\sum _{(q,s)\in Q^2} f_{layer,\zeta ,\eta } (q,s)[f_{layer,\zeta ,\eta } (q,s)-f_{layer} (q)f_{layer} (s)] \end{aligned}$$

**Figure 6:**
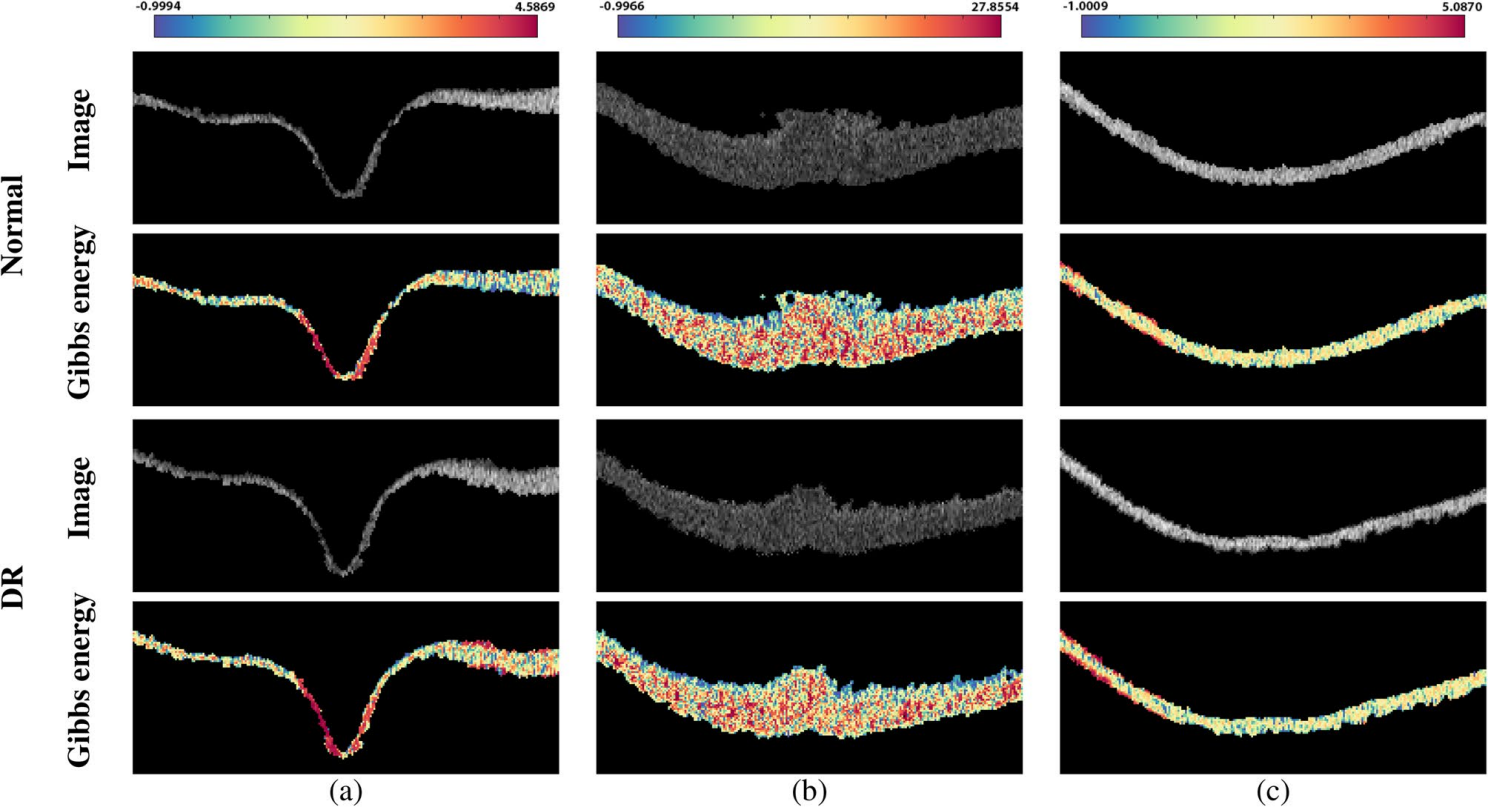
An illustrative color-coded example of the Gibbs energy for a normal (upper two rows) and a DR (lower two rows) case at three different layers: (**a**) NFL, (**b**) ONL, and (**c**) RPE layers.

Figure 6 shows a color-coded example of the estimated Gibbs energy at three different layers (NFL, ONL, and RPE) to demonstrate the discriminant difference between a normal and DR case. To summarize the 2nd-order reflectivity-based MGRF model, the basic steps are shown in Algorithm 1

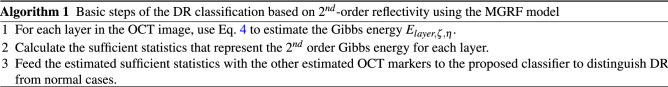



In addition to the local higher-order reflectivity term, a global higher-order reflectivity is also employed in our pipeline and is based on GLCM in which the values represent the number of times that pair of pixel intensities appear together in the 8-neighbors connectivity in the image. The constructed matrix is then normalized (the summation of all normalized values is 1, i.e., as a probability), then several statistical features can be extracted from it such as: *contrast*, *correlation*, *energy*, and *homogeneity*^44^. The contrast feature is measured using Eq. (5) which represents the difference of the intensity levels between a pixel and its neighbor. The correlation feature (Eq. 6) measures the correlation between a pixel and its neighbor. Energy (Eq. 7) shows the percent of the intensity differences. It ranges between 0 and 1, where 1 indicates that the image is fully homogeneous (i.e., only one grey level) and 0 means fully inhomogeneous. Finally, the homogeneity feature (Eq. 8) refers to the concentration of the GLCM elements around its diagonal. The range of its values is between 0 and 1.

**Figure 7:**
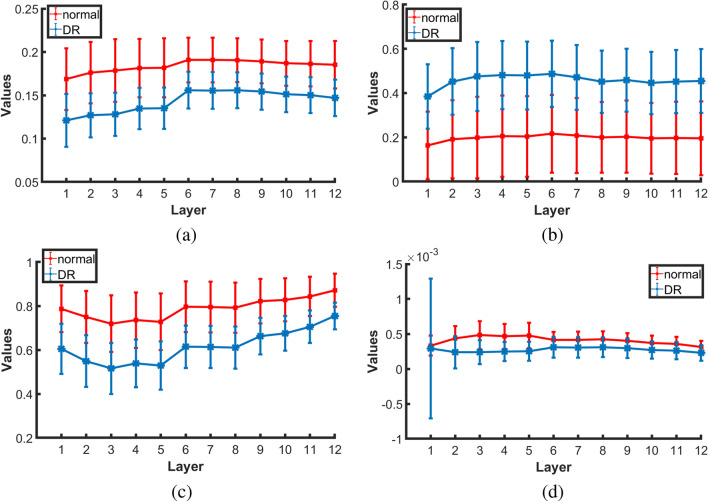
Average and standard deviation of the extracted GLCM features: (**a**) homogeneity, (**b**) contrast, (**c**) correlation, and (**d**) energy for all normal and DR cases at the twelve layers.

**Figure 8:**
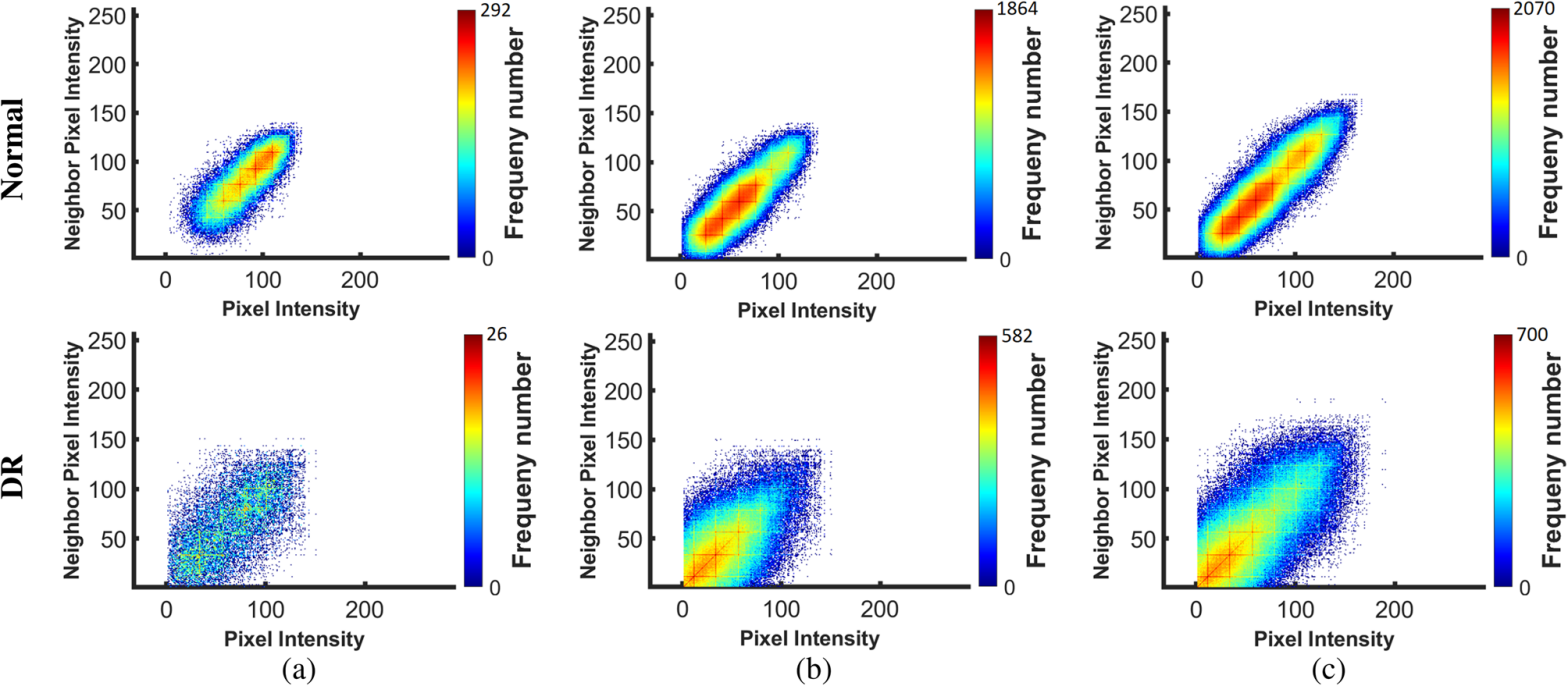
An illustrative color-coded example of GLCM features for a normal (upper row) and a DR (lower row) case at three different layers: (**a**) NFL, (**b**) ONL, and (**c**) RPE layers.

Figure 7 shows the average and standard deviation values of the extracted homogeneity, contrast, correlation, and energy markers in each layer for all normal and DR subjects. As demonstrated in the figure, there is an obvious difference between DR and normal subjects. Also, the color-coded maps of the GLCM elements are presented in Fig. 8 at three different layers (NFL, OPL, and RPE). As readily seen, the range of GLCM elements for DR classes is lower than the range in normal classes. Moreover, the global higher-order reflectivity markers are combined with the CDF percentiles of the 1st-order reflectivity as an individual reflectivity marker. Before the merger, 1st-order reflectivity for each layer is normalized by dividing each marker value of that layer by $95\%$ confidence interval for the mean of all its values for all cases in that layer, while each GLCM marker is normalized by dividing its values by the maximum number of that feature in all cases.

5 $$\begin{aligned} Contrast= & {} \sum _{i} \sum _{j} p(i,j) \left| i - j \right| ^2 \end{aligned}$$

6 $$\begin{aligned} Correlation= & {} \sum _{i} \sum _{j}\frac{p(i,j)(i-\mu _x)(j-\mu _y)}{\sigma _{x}\sigma _{y}} \end{aligned}$$

7 $$\begin{aligned} Energy= & {} \sum _{i} \sum _{j} p(i,j)^2 \end{aligned}$$

8 $$\begin{aligned} Homogeneity= & {} \sum _{i} \sum _{j}\frac{p(i,j)}{1+\left| i- j \right| } \end{aligned}$$

where

- *i* and *j* are the pixels pair of the GLCM;
- *p*(*i*, *j*) is the normalized probability of the GLCM elements
- $\mu _x = \sum _{i} \sum _{j} ip(i,j)$ and $\mu _y = \sum _{i} \sum _{j} jp(i,j)$ are the mean of the GLCM.
- $\sigma _{x} = \sqrt{\sum _{i} \sum _{j}(i-\mu _x)^2 p(i,j))}$ and $\sigma _{y} =\sqrt{\sum _{i} \sum _{j}(j-\mu _y)^2 p(i,j))}$ are the variance of GLCM.

**Figure 9:**
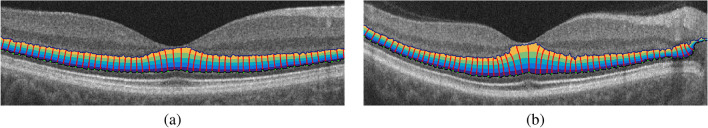
Two examples of the estimated point-wise correspondences for the ONL layer for a normal (**a**) and a DR (**b**) case.

#### Morphological features

The second set of markers that are used for DR diagnosis are the morphological markers. Those markers include the layer *thickness* and *tortuosity*. The *thickness* marker measures the thickness of the layer by estimating the Euclidean distance between two corresponding points on the layer boundaries. To avoid problems associated with signal intensity variations across retinal layers, we exploit a geometric approach instead to co-localize the point pairs^45^. Namely, the solutions of the Laplace equation allow for accurate localization of point-to-point correspondences between the boundaries of a segmented layer, as shown in Fig. 9. This second-order Laplace equation is defined as:

9 $$\begin{aligned} \nabla ^{2}\gamma =\frac{\partial ^{2}\gamma }{\partial x^{2}}+\frac{\partial ^{2}\gamma }{\partial y^{2}}=0 \end{aligned}$$

where $\gamma$ is the scalar field, or the harmonic function, that represents the estimated electric field between the upper and lower boundaries of the layer. The in-between intermediate equipotential surfaces and streamlines establish natural pixel-wise correspondences between the upper and lower boundaries of a given layer. In our work, we adopted a second-order central difference method and the iterative Jacobi approach to estimate $\gamma (x,y)$:

10 $$\begin{aligned} \gamma ^{i+1}(x,y)=\frac{1}{4}\biggl \{\gamma ^{i}(x+\Delta x,y)+\gamma ^{i}(x-\Delta x,y) +\gamma ^{i}(x,y+\Delta y)+\gamma ^{i}(x,y-\Delta y)\biggr \} \end{aligned}$$

where $\gamma ^{i}(x,y)$ is the estimated electric field at (*x*, *y*) during the $i{th}$ iteration; and $\Delta x$ and $\Delta y$ are the step length or resolution in *x* and *y* directions, respectively. An illustrative example of the estimated thickness for the ONL layer for a normal and a DR case is shown in Fig. 9. The estimated electrical field is represented by four color-coded ranges in which the blue (yellow) represents the highest (lowest) potential, as presented in the figure. Moreover, the starting points of each streamline with a constant distance is selected at the highest potential (i.e., green dots at the lower boundary). Then, select the low potential closest to each starting point to form its streamline. Also, the figure shows that there is an obvious difference between DR and normal subjects since the thickness of mid-region for the DR is larger than the normal case.

**Figure 10:**
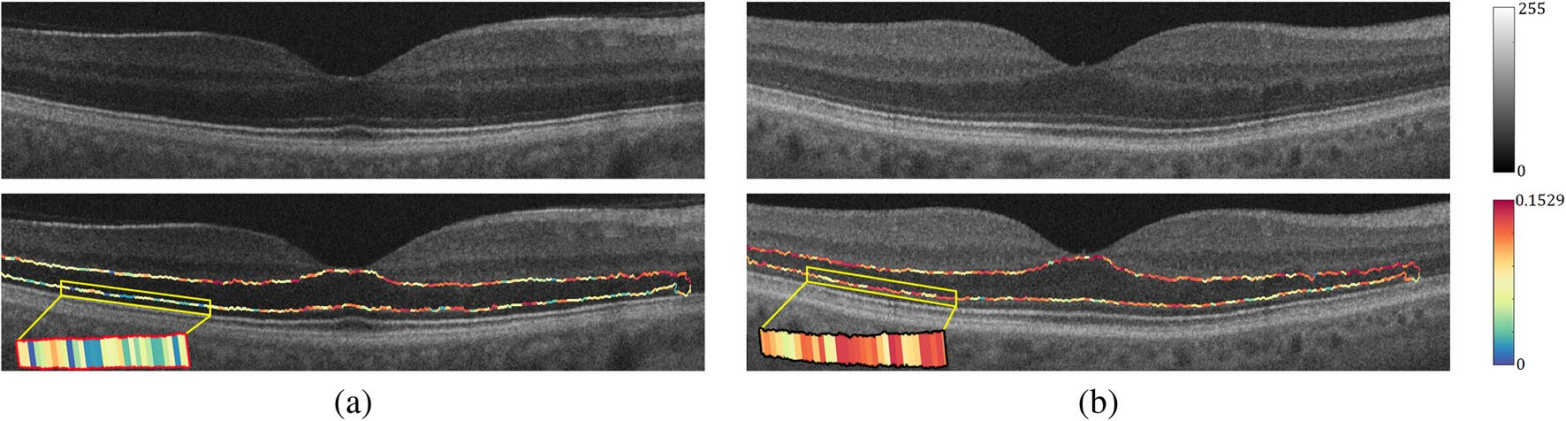
Color-coded examples of the estimated tortuosity for the ONL layer for (**a**) a normal and (**b**) a DR case.

On the other hand, the *tortuosity* (Eq. 11) of the layer boundary is measured by the absolute value of the local curvature. The latter is estimated using the multiplicative inverse radius of a circle passing through three sample points, $\left( x_1,y_1\right)$, $\left( x_2,y_2\right)$, $\left( x_3,y_3\right)$, on the boundary based on Menger curvature estimation^46^. An example of the estimated tortuosity for the ONL layer for a normal and a DR case is shown in Fig. 10. Moreover, the tortuosity descriptor of the layer is the combination of the tortuosity of the upper and lower boundaries of that layer.

11 $$\begin{aligned} \kappa =r^{-1} \end{aligned}$$

where $\kappa$ is the tortuosity estimated on a circle of radius *r* and center point $\left( x_0,y_0\right)$ which is solved using the following nonlinear system:

- $$\left( x_1-x_0\right) ^2+\left( y_1-y_0\right) ^2=r^2$$
- $$\left( x_2-x_0\right) ^2+\left( y_2-y_0\right) ^2=r^2$$
- $$\left( x_3-x_0\right) ^2+\left( y_3-y_0\right) ^2=r^2$$

### Classification

The classification method of the proposed system consists of three stages as demonstrated in Fig. 1: (1) An SVM with a linear kernel is fed with each extracted marker at each layer, individually. (2) Then, the DR probabilities resulting from the four classifiers at each layer are fed to a backpropagation neural network (BNN) with one hidden layer which involves 49 neurons to make the local diagnosis of that layer. (3) Finally, the outputs of the BNN of the twelve layers are fed to another BNN with 30 neurons in the hidden layer to make the final and global diagnosis of a given B-scan. The basic steps of the BNN are summarized in Algorithm 2

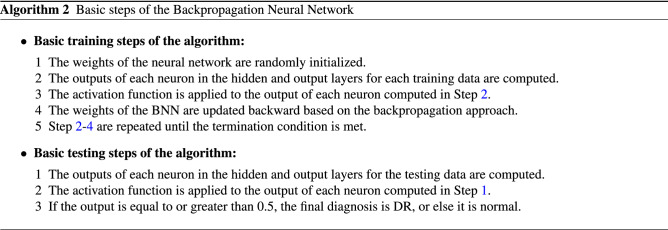



The hyper-parameters of the BNN are tuned by running different experiments only on $70\%$ of the dataset to overcome the overfitting problem. Those parameters include the number of hidden layers in the network, the number of neurons in the hidden layers, and the activation function used to estimate the output of each neuron. According to the conducted experiments, the best configurations to reach the results demonstrated in “Experimental results” are: one hidden layer (i.e., search space: 1–2) for all BNN used in the developed system; 49 and 30 neurons in the hidden layer for the local and the global diagnosis networks, respectively (search space: 1–50); and the activation functions applied to the hidden and output layers are hyperbolic tangent and softmax activation function, respectively (range: tangent, sigmoid, rectified linear, and softmax activation functions).

## Experimental results

The overall system assessment and evaluation is based on OCT data that are acquired using a Zeiss Cirrus HD-OCT 5000 machine^47^ from the Department of Ophthalmology and Visual Sciences at the University of Louisville (UofL) that have an axial resolution of 5 μm. Data collection and acquisition is certified by UofL Institutional Review Board (IRB) and the study adhered to the Declaration of Helsinki. The dataset includes 130 subjects with two scans for each eye (i.e., a total of 260 OCT images). The dataset is balanced and contains 65 subjects from each group (i.e., normal and DR). In our database, 79 cases (17 normal and 62 DR) have image resolution of $1024\times 350$ and 51 cases (48 normal and 3 DR) have image resolution of $1024\times 1024$, summarized in Table. 1. Grades of DR included in this study were mild nonproliferative diabetic retinopathy (NPDR) and moderate NPDR. Eyes with macula edema were not included in this study. Also, severe NPDR and proliferative diabetic retinopathy were not included because of low numbers of cases for each of those grades. These images were taken from a clinical setting with a minimum signal strength of 7/10 on the OCT machine in which all diabetic patients, regardless of ocular history, undergo macula OCT scans in each eye. The extent of disease and clinical grading was determined by physicians who were all trained retina specialists. They performed full dilated fundus exams to determine the extent of retinopathy in each eye.

**Table 1:** Dataset characteristic. Note that: OD and OS stand for right eye and left eye, respectively.

Class	Image size	Image type	OD	OS	Sub-total	Total
Normal	$1024\times 350$	Single-shot	17	17	34	130
	$1024\times 1024$	4x Averaged	9	9	18	
		20x Averaged	39	39	78	
DR	$1024\times 350$	Single-shot	62	62	124	130
	$1024\times 1024$	20x Averaged	3	3	6	

To present the performance of the developed system, *K*-fold cross-validation has been adopted for quantitative evaluation. In our study, different experiments have been conducted using different validation scenarios: 2-*folds*, 4-*folds*, 10-*folds*, and *leave-one-subject-out (LOSO)*. In addition, two experiments using different percentages of the dataset are conducted to demonstrate the optimization of the tuned BNN utilized in the integration between the individual markers. The results of these experimenters are reported in Table. 2. As demonstrated in the table, the accuracy of the developed system is improved when the size of the data is increased from $70\%$ (i.e., 180 cases) to $100\%$ of the dataset (i.e., all the 260 cases). Quantitative performance has been measured using different metrics: *sensitivity*, *specificity*, *F1-score*, and *accuracy* estimated as indicated in Table  3.

**Table 2:** The classification accuracy of the proposed BNN-based system at different sizes of the dataset.

	Dataset percentage	Sensitivity	Specificity	F1-score	Accuracy
2 Folds	$70\%$	$91.02\%\pm 2.6\%$	$91.07\%\pm 12.6\%$	$89.04\%\pm 2.6\%$	$89.44\%\pm 0.8\%$
	$100\%$	$93.94\%\pm 1.0\%$	$97.30\%\pm 3.8\%$	$95.15\%\pm 1.9\%$	$95.38\%\pm 1.1\%$
4 Folds	$70\%$	$93.54\%\pm 7.5\%$	$93.58\%\pm 7.8\%$	$91.86\%\pm 1.5\%$	$92.22\%\pm 1.3\%$
	$100\%$	$94.62\%\pm 4.4\%$	$98.65\%\pm 2.7\%$	$96.33\%\pm 1.4\%$	$96.54\%\pm 0.8\%$
10 Folds	$70\%$	$93.83\%\pm 8.5\%$	$100\%\pm 0.0\%$	$96.64\%\pm 4.6\%$	$96.11\%\pm 5.3\%$
	$100\%$	$96.75\%\pm 5.5\%$	$96.41\%\pm 4.9\%$	$96.82\%\pm 3.2\%$	$96.54\%\pm 3.8\%$
LOSO	$70\%$	95.45%	95.65%	95.45%	95.56%
	$100\%$	96.15%	99.23%	97.66%	97.69%

**Table 3:**
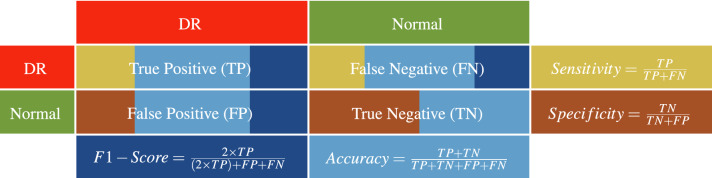
Performance metrics used for the diagnostic evaluation of the proposed system. *TP* (*TN*) is the number of times identifying the DR (normal) classes correctly, while *FN* (*FP*) is the number of times identifying the DR (normal) classes incorrectly.

To highlight the promise of the integrated markers in the proposed system, the performance of the system is assessed using individual markers separately. The obtained results are based on using an SVM to classify the extracted feature from each layer, and then these results are fused using a one hidden layer BNN with 30 neurons. The marker-wise evaluation is demonstrated in Table 4. As shown in the table, the reflectivity marker gives the highest performance compared with the other three markers. One of the contributions of this study is to enhance the accuracy of the system by fusing different markers. This is confirmed by the results in Table 4 as the performance of the proposed system is enhanced by $2\%$ over the highest accuracy of the four markers using LOSO validation. The proposed system achieved $96.15\%$, $99.23\%$, $97.66\%$, and $97.69\%$ for the sensitivity, specificity, F1-score, and accuracy, respectively. It is worth mentioning that the markers fusion, combined with LOSO, gives the best performance in all cross-validating techniques.

**Table 4:** The classification accuracy of the proposed BNN-based system compared with the performance of each marker separately.

	Features	Sensitivity	Specificity	F1-score	Accuracy
2 Folds	Gibbs energy	$86.53\%\pm 3.9\%$	$71.04\%\pm 10.6\%$	$79.49\%\pm 6.1\%$	$78.08\%\pm 4.9\%$
	Thickness	$84.75\%\pm 1.4\%$	$76.42\%\pm 10.6\%$	$80.55\%\pm 7\%$	$80\%\pm 5.4\%$
	Tortuosity	$92.13\%\pm 6.1\%$	$71.26\%\pm 12.9\%$	$82.46\%\pm 5.9\%$	$80.77\%\pm 5.4\%$
	Reflectivity	$90.35\%\pm 3.5\%$	$100\%\pm 0\%$	$94.91\%\pm 2\%$	$95\%\pm 2.7\%$
	BNN-Based Fusion	${93.94\%\pm 1.0\%}$	${97.3\%\pm 3.8\%}$	${95.15\%\pm 1.9\%}$	${95.38\%\pm 1.1\%}$
4 Folds	Gibbs energy	$85.92\%\pm 13.2\%$	$72.99\%\pm 9.8\%$	$79.78\%\pm 8.5\%$	$78.85\%\pm 8\%$
	Thickness	$79.15\%\pm 7.2\%$	$79.21\%\pm 13\%$	$78.67\%\pm 10.1\%$	$79.23\%\pm 8.6\%$
	Tortuosity	$95.47\%\pm 3.9\%$	$72.32\%\pm 12.7\%$	$85.04\%\pm 7.9\%$	$83.46\%\pm 8.5\%$
	Reflectivity	$92.37\%\pm 5.9\%$	$96.86\%\pm 2.3\%$	$94.44\%\pm 4.5\%$	$94.62\%\pm 3.9\%$
	BNN-based fusion	${94.62\%\pm 4.4\%}$	${98.65\%\pm 2.7\%}$	${96.33\%\pm 1.4\%}$	${96.54\%\pm 0.8\%}$
10 Folds	Gibbs energy	$91.36\%\pm 7.4\%$	$71.73\%\pm 21.8\%$	$83.43\%\pm 9.3\%$	$81.92\%\pm 10.4\%$
	Thickness	$79.59\%\pm 15.7\%$	$74.27\%\pm 22.9\%$	$77.39\%\pm 15\%$	$78.08\%\pm 12.4\%$
	Tortuosity	$95.91\%\pm 5.6\%$	$73.67\%\pm 15.9\%$	$85.95\%\pm 10\%$	$84.62\%\pm 10.1\%$
	Reflectivity	$92.94\%\pm 8.3\%$	$96.7\%\pm -5.7\%$	$94.66\%\pm 5.8\%$	$94.62\%\pm 5.8\%$
	BNN-based fusion	${96.75\%\pm 5.5\%}$	${96.41\%\pm 4.9\%}$	${96.82\%\pm 3.2\%}$	${96.54\%\pm 3.8\%}$
LOSO	Gibbs energy	86.92%	71.54%	80.71%	79.23%
	Thickness	83.85%	71.54%	78.99%	77.69%
	Tortuosity	94.62%	73.85%	85.71%	84.23%
	Reflectivity	90.77%	100.00%	95.16%	95.38%
	BNN-Based Fusion	96.15%	99.23%	97.66%	97.69%

Note that: BNN and LOSO stand for backpropagation neural network and leave-one-subject-out, respectively.

**Figure 11:**
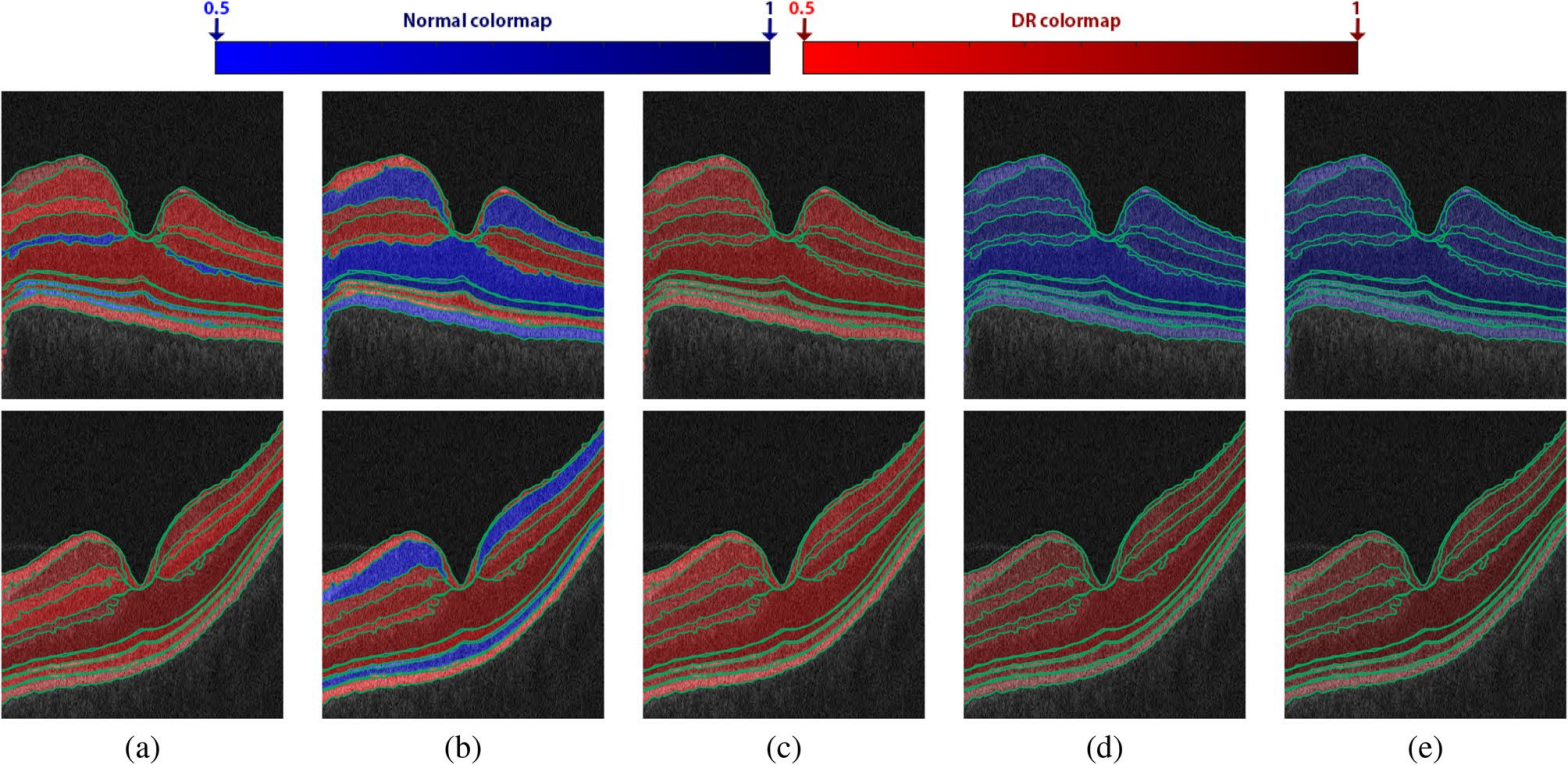
Illustrative color-coded map examples of all retinal layers’ classification for a healthy retina (upper row) and a DR case (lower row) for individual markers: (**a**) Gibbs energy, (**b**) thickness, (**c**) tortuosity, (**d**) reflectivity, and (**e**) their fusion. The range of the color maps consists of 100 different colors for both normal (50 blue) and DR (50 red) classes to visually represent the probability of the classification from 0.5 to 1 with 0.1 increments.

Moreover, to highlight the advantage of the marker fusion, the probability of the resulting classification at each layer is visually represented using color-coded maps using blue and red hues for the normal and DR cases, respectively. The light color represents the lower probability, and vice versa. The decision of classification is made by selecting the class that has a higher probability. Figure 11 shows an example of the color-coded maps for a normal and DR case. As shown in the figure, reflectivity and the fused markers classify each layer correctly. However, if one looks carefully, the color maps of the fused markers are darker than the reflectivity, indicating greater intra-class coherence than in the reflectivity example. This confirms that the developed system distinguishes DR and normal classes much better than individual markers.

**Table 5:** Classification accuracy of the proposed BNN-based fusion system compared with other different fusion approaches.

	Classifiers	Sensitivity	Specificity	F1-Score	Accuracy
2 Folds	SVM + MV	91.02% ± 2.6%	98.65% ± 1.9%	94.42% ± 0.2%	94.62% ± 1.1%
	SVM + SVM	91.02% ± 2.6%	95.27% ± 6.7%	92.36% ± 2.7%	92.69% ± 1.6%
	MV + SVM	90.35% ± 3.5%	88.51% ± 16.2%	88.36% ± 7.3%	88.46% ± 6.5%
	MV + MV	87.64% ± 7.4%	89.19% ± 15.3%	87.12% ± 4.6%	87.31% ± 3.8%
	BNN + MV	92.13% ± 6.1%	95.95% ± 5.7%	93.31% ± 0.3%	93.46% ± 0.5%
	MV + BNN	89.45% ± 2.3%	91.00% ± 10.2%	89.29% ± 5.1%	89.62% ± 3.8%
	BNN + SVM	93.48% ± 4.2%	90.54% ± 13.4%	91.07% ± 5.6%	91.15% ± 4.9%
	SVM + BNN	91.70% ± 1.6%	97.97% ± 2.9%	94.36% ± 0.9%	94.62% ± 0%
	Proposed (BNN + BNN)	93.94% ± 1.0%	97.30% ± 3.8%	95.15% ± 1.9%	95.38% ± 1.1%
4 Folds	SVM + MV	91.68% ± 6.1%	100% ± 0%	95.58% ± 3.3%	95.77% ± 2.9%
	SVM + SVM	93.03% ± 6%	97.72% ± 2.7%	95.14% ± 4.6%	95.38% ± 3.8%
	MV + SVM	91.02% ± 3.6%	92.99% ± 8.3%	91.33% ± 5.4%	91.54% ± 4.8%
	MV + MV	90.32% ± 6.7%	89.00% ± 12.1%	88.71% ± 7.7%	88.85% ± 7%
	BNN + MV	91.64% ± 7.0%	100% ± 0%	95.54% ± 3.8%	95.77% ± 3.4%
	MV + BNN	90.32% ± 6.7%	90.97% ± 8.7%	89.71% ± 5.6%	90.00% ± 4.6%
	BNN + SVM	94.82% ± 3.7%	96.37% ± 3.4%	95.28% ± 0.7%	95.38% ± 0%
	SVM + BNN	94.82% ± 3.7%	96.8% ± 3.1%	95.8% ± 1.7%	95.77% ± 1.9%
	Proposed (BNN + BNN)	94.62% ± 4.4%	98.65% ± 2.7%	96.33% ± 1.4%	96.54% ± 0.8%
10 Folds	SVM + MV	92.31% ± 7.9%	100% ± 0%	95.84% ± 4.4%	95.77% ± 4.2%
	SVM + SVM	93.65% ± 8.6%	96.23% ± 7.9%	95.03% ± 5.6%	95.00% ± 6%
	MV + SVM	92.43% ± 9%	93.02% ± 15.4%	93.47% ± 5.8%	93.08% ± 7.2%
	MV + MV	90.88% ± 9.6%	91.95% ± 10.8%	90.44% ± 7.4%	90.77% ± 6.6%
	BNN + MV	93.77% ± 7.6%	100% ± 0%	96.64% ± 4.2%	96.54% ± 4.2%
	MV + BNN	91.6% ± 8.2%	93.15% ± 12.1%	92.3% ± 5.8%	92.31% ± 6%
	BNN + SVM	96.75% ± 5.5%	96.79% ± 5.4%	96.69% ± 3.9%	96.54% ± 4.2%
	SVM + BNN	93.65% ± 8.6%	98.75% ± 4%	96.26% ± 4.9%	96.15% ± 5.1%
	Proposed (BNN + BNN)	96.75% ± 5.5%	96.41% ± 4.9%	96.82% ± 3.2%	96.54% ± 3.8%
LOSO	SVM + MV	91.54%	100.00%	95.58%	95.77%
	SVM + SVM	93.08%	97.69%	95.28%	95.38%
	MV + SVM	92.31%	93.08%	92.66%	92.69%
	MV + MV	90%	93.08%	91.41%	91.54%
	BNN + MV	90.77%	100%	95.16%	95.38%
	MV + BNN	90.77%	93.85%	92.19%	92.31%
	BNN + SVM	96.92%	98.46%	97.67%	97.69%
	SVM + BNN	92.31%	98.46%	95.24%	95.38%
	Proposed (BNN + BNN)	96.15%	99.23%	97.66%	97.69%

The left and the right sides of the “$+$” in the second column indicate the fusion approach for four markers at each layer and the fusion approach of the twelve layers to make the final decision, respectively. Note that: SVM, MV, BNN, and LOSO stand for support vector machine, majority voting, backpropagation neural network, and leave-one-subject-out, respectively.

**Table 6:** Classification accuracy of the proposed system compared with different machine learning classifiers.

	Classifiers	Sensitivity (%)	Specificity (%)	F1-score (%)	Accuracy (%)
LOSO	NB	92.31	96.92	94.49	94.62
	RF	93.08	97.69	95.28	95.38
	KNN	92.31	99.23	95.62	95.77
	DT	94.62	90.00	92.48	92.31
	Proposed System	96.15	99.23	97.66	97.69

Note that: NB, RF, KNN, DT, SVM, and LOSO stand for Naïve Bayes, random forest, K-nearest neighbors, decision tree, support vector machine, and leave-one-subject-out, respectively.

**Figure 12:**
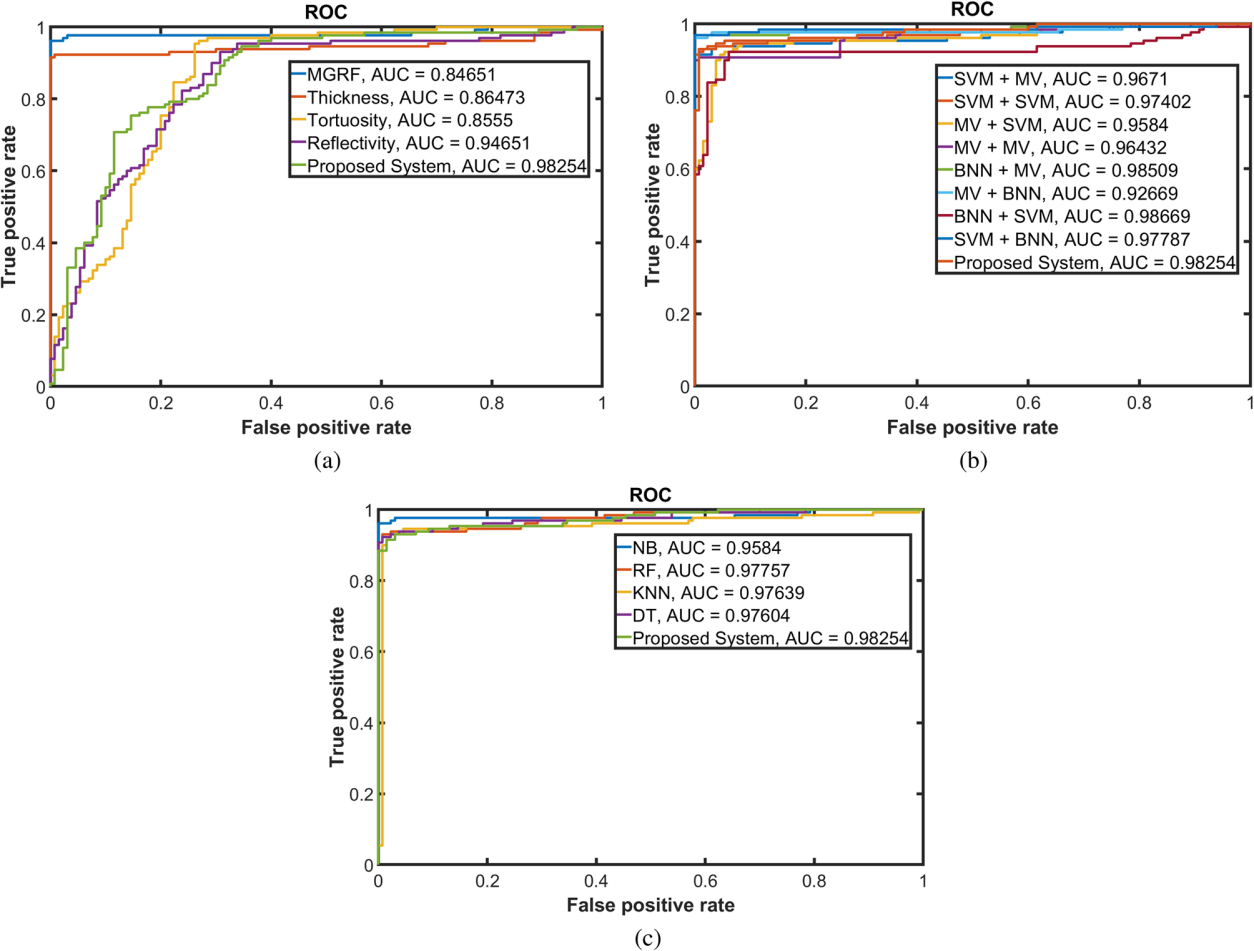
Receiver operating characteristic (ROC) curves of the developed system in comparison with classification obtained using (**a**) individual markers; (**b**) different fusion approaches; and (**c**) different statistical ML. The area under the ROC curve (AUC) of the proposed system is $98.25\%$.

To the best of our knowledge, there is limited literature for DR diagnosis using OCT. Most of recent work has proposed systems using FP images or using CNNs^8,9^. These two environments are different from our proposed system, and it would be unfair to compare our system with them. Therefore, to show more of the virtues of the developed system, two more experiments have been conducted and compared with the developed system, as demonstrated in Tables 5 and 6. Table 5 demonstrates the results obtained using different fusion approaches at both the markers level (Level 1) and the layers level (or Level 2). Particularly, instead of using two BNNs as fusion methods at levels 1 and Level 2, an SVM with a linear kernel and a majority voting (MV) fusion methods are used and their performance is compared with the current proposed system (see Table 5). The configuration of the BNNs for the results demonstrated in Table 5 is one hidden layer with 49 and 30 neurons for Level 1 and Level 2 fusion. As shown in the table, the performance of the developed system is the highest compared with other fusion approaches. Although, the accuracy of (BNN $+$ MV) and (BNN $+$ SVM) scenarios is equal to that of the proposed (BNN $+$ BNN) system in 10-folds cross-validation, their variations (standard deviation values) are higher, which means that the proposed system is more stable. Also, the accuracy of the (BNN + MV) approach is equal to the accuracy of the developed system in LOSO cross-validation. Since one of the two fusion methods in the (BNN + MV) experiment is a BNN, which is a part of the proposed system, it is logic to give the same performance in some experimental trials. Overall, the proposed system is much better than the other approaches as shown in Table 5.

To more prove the stability of the system, different machine learning (ML) classifiers have been tested instead of SVM and their performance is compared with the developed system. The results are summarized in Table 6. From the table, the performance of the developed system is the highest if compared with other ML classifiers, which supports the stability of the proposed system.

Finally, to show the robustness of our approach to distinguish between normal and DR cases for the three conducted experiments, receiver operating characteristic (ROC) curves have been constructed, see Fig. 12. The AUC of the proposed system is $98.25\%$ which is higher than all the experiments except (BNN + MV) and (BNN + SVM) fusion approaches whose AUCs are $98.51\%$ and $98.67\%$, respectively. These AUCs are very close to our proposed system’s AUC. This can be explained in part by the fact that the Level 1 fusion of these two experiments is the same as the Level 1 fusion of the proposed system. All of the above experiments confirm that BNN is one of the best approaches to fuse between different results to produce the final decision in this proposed system, thus making it highly accurate to diagnose DR using OCT B-scans.

Clinical retina specialists can easily identify DR in the clinical setting, we foresee the ultimate use of this technology as one of screening. Because all diabetics need to be screened for diabetic retinopathy at least annually, automated screening with imaging modalities, such as OCT, is the way forward.

## Conclusions and future works

In this study, a new CAD system was presented to detect DR early using non-invasive OCT B-scans. The system estimates different discriminant morphology and reflectivity markers from automatically segmented retinal layers. These descriptors are fused and classified using current, state-of-the-art machine learning classifiers. The best accuracy of these descriptors was $95.38\%$, improving to $97.69\%$ with the integration of these descriptors. Moreover, these results show the advantage of integrating discriminate markers using BNN, rather than different fusion approaches in diagnosing DR. In the future, we plan to investigate and study the enhancement of the proposed system in combination with other scan modalities as well as the clinical biomarkers. Moreover, this system can be generalized to diagnose different eye pathologies that affect the retinal layers and cause vision loss.

## Data Availability

Materials, data, and associated protocols will be available to readers after the manuscript gets accepted.
